# Synchrony 2022: Catalyzing Research and Treatments to Benefit Individuals with Neurodevelopmental Disorders including Autism Spectrum Disorders

**DOI:** 10.3390/jpm13030490

**Published:** 2023-03-09

**Authors:** Heer Nanda, Richard E. Frye

**Affiliations:** 1 University of California, Berkeley, CA 94720, USA; 2 Autism Discovery and Treatment Foundation, Phoenix, AZ 85050, USA; 3 Rossignol Medical Center, Phoenix, AZ 85050, USA; 4 Southwest Autism Research and Resource Center, Phoenix, AZ 85006, USA

## 1. Introduction

A unique translational medicine conference for research into treatments that can benefit individuals with neurodevelopmental disorders (NDD), including autism spectrum disorder (ASD), has been developed and hosted by The BRAIN Foundation (Pleasanton, CA, USA) since 2019. Synchrony 2022, which occurred over three days in December 2022, brought together both basic and clinical sciences from both academia and industry to expedite the translation of ASD research, not only from bench to bedside but also from the developmental stages in academia to commercialization in industry. Physicians were able to take advantage of CME-accredited training to bring this knowledge to the fingertips of physicians who interact with patients every day. This international conference provided a forum for world-renowned scientists and biotech and pharmaceutical companies to share their research on developing FDA-approved treatments for those who suffer from impairments caused by ASD and other developmental disabilities.

The conference occurred over three days and was divided into three primary sections to facilitate the cross-pollination of ideas between researchers, clinicians, and industry leaders, allowing the facilitation and advancement of research into ASD and the identification of knowledge gaps that need to be solved in order to advance the field. In academic sessions, established academic scientists pursuing some of the most promising cutting-edge research presented their research along with additional presentations by new, young investigators just starting in the field. In the industry sessions, scientists from companies developing treatments for ASD provided insight into their unique therapeutic approaches. Four roundtables brought together academic researchers, clinicians, private practice clinicians, industry partners, and the parents of children with ASD to discuss some of the most difficult and unsolved problems in ASD.

The researchers and scientists who attended this conference included those from well-known institutions such as Stanford, the University of California, San Francisco, Harvard, Brown University, and others. The scientific advisory board at the BRAIN Foundation consisted of highly talented and skilled scientists, including Sarkis Mazmanian (Ph.D.)—Luis and Nelly Soux, Professor of Microbiology in the Division of Biology and Biological Engineering at Caltech (Pasadena, CA, USA), Paul Ashwood (Ph.D.)—Professor of Microbiology and Immunology at the MIND Institute at the University of California, Davis (Davis, CA, USA), Alessio Fasano (MD)—Chief of Pediatric Gastroenterology and Nutrition at Massachusetts General Hospital (Boston, MA, USA), Richard E Frye (MD, Ph.D.)—President of the Autism Discovery and Treatments Foundation (Phoenix, AZ, USA), James Adams (Ph.D.)—Professor at Arizona State University (Tempe, AZ, ISA), and Randy Blakely (Ph.D.)—Executive Director at the Stiles-Nicholson Brain Institute at Florida Atlantic University (Jupiter, FL, USA), most of whom are currently pursuing research into the causes and treatments for ASD. The meeting format also intentionally provided many opportunities for networking between the stakeholders while inviting community members for a meeting and greet dinner with participants. A pre-conference retreat to a nearby wine country was included to facilitate interaction and discussions. This editorial summarizes the new findings on ASD that were presented during the Symposium.

## 2. Roundtables and Working Groups

The conference hosted four very productive roundtables. Dr. Jennifer Frankovich (MD), Clinical Professor at the Department of Pediatrics at Stanford Children’s Health (Palo Alto, CA, USA), chaired a roundtable concerning the potential role of neuroinflammation in ASD. The panel found that this was a substantially under-researched area that had much promise. The panel decided that the most practical immediate application for the potential role of neuroinflammation was to develop a consensus article aimed at guiding the clinician in the investigation and management of acute behavioral exacerbations in individuals with ASD, where neuroinflammation could have a role.

Dr. Gabriel Belfort (MD, Ph.D.), Vice President at Axial Therapeutics Incorporation (Woburn, MA, USA), chaired a roundtable that focused on the pathways and the challenges of obtaining medications that were FDA-approved. The challenges with developing new drugs and devices through the FDA pipeline as well as the challenges with designing clinical trials and outcome measures as the main point of discussion.

Mr. John Slattery, Co-Founder, President, and CEO of BioROSA Technologies, Inc. (Watertown, MA, USA), led a roundtable focusing on the potential biomarkers that are used to guide the diagnosis and treatment of ASD. The panel identified many gaps and challenges in the development of biomarkers for the diagnosis and guidance of treatment for ASD. The panel decided to create a consensus paper reviewing some of the most promising biomarkers and defining the best practices for developing biomarkers for ASD.

Dr. Richard E Frye (MD, Ph.D.), President of the Autism Discovery and Treatments Foundation (Phoenix, AZ, USA), chaired an all-day roundtable session that included six presentations by leaders in the field of ASD and epilepsy as well as parent presentations describing their challenges with controlling seizures in their children with ASD. Dr. Samuel J Pleasure described his work on autoimmunity and epilepsy, Dr. Manuel F Casanova discussed the neuropathology of ASD and epilepsy, Dr. Jeffrey Lewine described his work using magnetoencephalography to study epilepsy and subclinical epileptiform discharges in ASD, Dr. John Gaitanis discussed treatment approaches for refractory epilepsy in ASD, Dr. James B Adams discussed nutritional and vitamin support in epilepsy and ASD, Dr. Richard G Boles discussed the contribution of genetic variants in the etiology of epilepsy in ASD and Dr. Richard E Frye discussed metabolic disorders related to ASD and epilepsy. The group developed a list of multiple topics that needed to be better defined in ASD and epilepsy and developed a plan for publishing multiple papers, including a guideline for the workup and management of individuals with ASD and epilepsy. 

## 3. Research Presentations

The research presentations were opened by Pramila Srinivasan (Ph.D.), President and Founder of The BRAIN Foundation. Presentations covered topics from serotonin, microglia, novel drug targets, gut microbial metabolites, multi-omics, and more. These presentations were selected from among numerous submissions to our call for abstracts earlier in the year. A total of seven talks were chosen for presentation. Two of the speakers were awarded the Most Innovative Research and Most Impactful Research, which included a sum of $15,000 each.

Randy Blakely (Ph.D.), Executive Director at the Stiles-Nicholson Brain Institute at Florida Atlantic University (Jupiter, FL), discussed his research on the disrupted, bidirectional relationship between serotonin and inflammatory signaling that arises in mice expressing a hyper-functional, ASD-associated variant in the serotonin transporter (SERT Ala56). His research has revealed that the mutant mice demonstrate hyperserotonemia: a well-replicated ASD biomarker that is enhanced with serotonin clearance in the CNS elevated p38α MAPK-dependent SERT phosphorylation, and behavioral traits such as changes in social communication and repetitive behavior, as well as gut phenotypes. As p38α MAPK activation and the upstream inflammatory cytokine IL-1b could elevate SERT function, Blakely proposed that the high level of expression of the IL-1b receptor (IL-1R1) provides a pathway by which inflammation constitutively upregulates SERT and the effects mimicked by the SERT Ala56 mutant [1].

Naveen Nagarajan (Ph.D.), a researcher at the University of Utah (Salt Lake City, UT, USA), discussed microglia-specific circuit defects in repetitive ASD behaviors. From his research, he found that the loss of function in the microglia Hoxb8 gene leads to repetitive grooming behaviors and chronic anxiety, leading to cortico-striatal circuit defects. This is significant as this suggests that defects in microglia can alter the function of neural circuits, leading to ASD-related behaviors [2].

Stephen E.P. Smith (Ph.D.), an Associate Professor at the University of Washington (Seattle, WA), presented on the identification of novel drug targets through the analysis of protein interaction network dysfunction. He used quantitative multiplexed co-immunoprecipitation to measure how the mutation of the ASD-linked protein FMR1 alters activity-dependent synaptic protein–protein interaction networks in mice [3]. The SRC-family kinase FYN was the most dysregulated network node, and inhibition of SRC-family kinases led to the normalization of molecular and behavioral phenotypes, suggesting that FYN manipulation may be a therapeutic option for Fragile X [3].

Brittany Needham (Ph.D.), a Faculty Member at the Stark Neurosciences Research Institute at Indiana University School of Medicine (Indianapolis, IN, USA), talked about how gut microbial metabolites influence the brain and behavior in a preclinical and clinical ASD-based context. While studying fecal and plasma samples of mice with atypical neurodevelopment (ASD mouse models), the microbial metabolite 4-ethylphenyl sulfate (4EPS), which is produced in the gut, was found at high levels when compared to the controls. When 4EPS entered the brain, it was found that it impaired the oligodendrocyte maturation and oligodendrocyte-neuron interaction, reducing the myelination of neural axons, which is commonly associated with anxiety-like behaviors [4].

Maude David (Ph.D.), an Assistant Professor at Oregon State University (Corvallis, OR, USA), presented her multi-omics, microbiome, and behavioral studies and their importance in relation to fatty acid metabolism in ASD. She conducted a longitudinal study on siblings 2 years apart, one with and one without ASD, by collecting their stool samples 0, 2, and 4 weeks into the study and comparing them to their lifestyle, behavior, and diet as it relates to the microbial structure [5]. 

Richard G Boles (MD), a Director of the NeuroGenomics Program at NeurAbilities Healthcare (Township, NJ, USA), presented on the high sensitivity for monogenic casual diagnoses in ASD, including de novo variants that represented novel disorders, with trio whole genome sequencing and data reanalysis. He studied DNA sequencing for 20 patients with trio whole genome sequencing and found that 15 of the cases had monogenic causal diagnoses while 16 of the cases had treatment recommendations based on DNA sequencing results. This shows that re-analyzing DNA sequencing files greatly enhances the yield of trio whole genome sequencing for revealing molecular diagnoses in ASD and that there are many overlapping genes for ASD and epilepsy [6]. 

Benjamin Marlow (MBiochem, MBBS, MRCPCH, PGCME), a Consultant Pediatrician at Re:Cognition Health (RCH) (Merrifield, VA, USA), presented on the research success in Alzheimer’s to make an impact in the field of autism. RCH works on creating medicinal products, identifying biomarkers, and studying patients with Alzheimer’s for comparison to those with ASD. At RCH, scientists have found similar biological mechanisms in those with Alzheimer’s to certain ASD phenotypes as they are related to immune dysfunction, and there is hope that this could be used as a starter to identify biomarkers and research novel therapeutics in the area of ASD.

## 4. Invited Speakers

The Invited Speaker’s session on the last day was the most widely attended session by the general public. This year, the session was opened with an address by Pramila Srinivasan, Ph.D., Founder and President of the BRAIN Foundation. She talked about the main accomplishments of the BRAIN Foundation, including the funding of research that had totaled over $1M in the past year. Other activities, including industry support and community support and workshops, and other activities, were highlighted. Goals for the next year were presented as well.

Manish Arora (BDS, MPH, Ph.D.) and Edith J Baerwald, both Professor and Vice Chairman of the Department of Environmental Medicine and Public Health, Icahn School of Medicine at Mount Sinai (New York, NY), presented topics on harnessing the human exposome to diagnose ASD. By analyzing hair samples over time, Dr. Arora described how an environmental biodynamic approach could help characterize time-dependent changes in thousands of chemical compounds deposited in the hair and provide information to accurately detect ASD early on in life.

Richard E Frye (MD, Ph.D.), President of the Autism Discovery and Treatment Foundation (Phoenix, AZ, USA), talked about his new findings on mitochondrial research. Mitochondrial disease is rarely found in the general population but is found at high rates in those who have ASD (~5%). While conducting research studies, Dr. Frye found that some children with ASD, particularly those with neurodevelopmental regression, experienced higher than normal respiration rates and a higher reserve capacity when compared to those without ASD. Additionally, this subset of those with ASD presented morphological differences in their mitochondria. Dr. Frye then showed data that linked this unique abnormality in mitochondrial respiration to prenatal exposure to air pollution and nutritional metals such as zinc and manganese [7].

T. Atilla Ceranoglu (MD) from Massachusetts General Hospital (Boston, MA) studied the evaluation of transcranial photobiomodulation in ASD. Transcranial photobiomodulation (tPBM) utilized a specific wavelength of near-infrared light that penetrated into the brain tissue and stimulated the mitochondria, leading to increased cellular metabolism and gene transcription. tPBM has been associated with a reduction in proinflammatory cytokines, a reduction in the amount of activated immune cells, and stimulation neurogenesis and neuroprotection. To test out tPBM, an open-label single-group design was conducted, and the results were positive. Most of the patients had a 25% decrease in SRS-2 scores and CGI-I, with several indicating a 10-point increase in GAF scores [8].

John Gaitanis (MD), a Pediatric Neurologist and Epileptologist at Hasbro Children’s Hospital (Providence, RI), presented the interplay between medical cannabis and ASD. Cannabidiol has many positive effects on those with ASD, including improving sleep and anxiety management and positively affecting oxytocin and serotonin levels in the body, which can lead to an increase in social interaction. One report found that using pure CBD and pure THC in certain dosages had a 49% improvement rate in the overall behavior of those with ASD [9].

Alessio Fasano (MD), Director of the Mucosal Immunology and Biology Research Center at Massachusetts General Hospital for Children (Boston, MA, USA), talked about the evaluation of the zonulin pathway for the personalized treatment of ASD using a humanized mouse model and human intestinal tissue. Zonulin is a pre-haptoglobin-2 protein (pre-HP2) that is known to regulate intestinal permeability and is stimulated by gut dysbiosis. The results of his studies showed that when ASD patients experienced gastrointestinal symptoms, there was an overrepresentation of HP2-2 and an underrepresentation of HP1-1. Additionally, the Ztm HP2-2 mice that received the human ASD fecal microbial transplant, compared to the wild-type HP1-1 mice that received the control fecal microbial transplant, exhibited different behaviors [10].

Arthur Krigsman (MD), a Pediatric Gastroenterologist at a Private Practice (New York, NY, USA), and Stephen Walker (Ph.D.), a Professor at the Wake Forest Institute for Regenerative Medicine (Winston-Salem, NC, USA), presented on the improvement in gastrointestinal symptoms, cognition, and behavior upon the treatment of ASD-associated enterocolitis. Even though ASD-enterocolitis is distinct from Crohn’s disease, there is a lot of cellular and molecular overlap; hence, they suggested that treating those who have ASD-associated enterocolitis the same as any other patient who has Crohn’s disease could have positive results. While patients were receiving treatment during studies, ASD questionnaires, GI symptom trackers, and behavior scales were used to measure improvement, and within the first few weeks of treatment, there was a significant improvement in producing bowel movements. As this cohort was measured for 52 weeks, there was an overall significant improvement in both the gastrointestinal and ASD symptoms [11].

Joseph D. Buxbaum (Ph.D.), a Professor of Psychiatry, Neuroscience, and Genetics and Genomic Sciences at the Icahn School of Medicine at Mount Sinai (New York, NY, USA), talked about the rare mutations and common treatments for ASD. A total of 350 genes have been found to have mutations that can lead to ASD. Using CRISPRi (made by coupling CRISPR-Cas9 transcription), he has found the functional signatures for 77 of the main ASD risk genes. Through this technology and studies, it was found that several ASD-risk genes could delay or accelerate neural differentiation. He is now attempting to match gene expression patterns with existing drug signatures to potentially detect and work on treating ASD early [12].

James B Adams (Ph.D.), President Professor at Arizona State University (Tempe, AZ, USA), talked about microbiota transplant therapy for Pitt–Hopkins syndrome. Pitt–Hopkins syndrome is the mutation or deletion of the Tcf4 gene on chromosome 18 and typically leads to very severe ASD, intellectual disability, and physical disability. He and his collaborator Prof. Rosa Krajmanlnik-Brown conducted a clinical trial for microbiota transplant therapy for Pitt–Hopkins syndrome and found that there was an improvement in ASD symptoms and a significant reduction in gastrointestinal and other symptoms. Additionally, utilizing studies of vitamin/mineral/micronutrient supplementation for ASD, Dr Adams was able to use the results to create a product called ANRC-Essentials Plus (ANRC-EP), which is now being used for a wide range of children and adults with ASD [13].

Harumi Jyonouchi (MD), a Clinical Professor of Pediatrics at Rutgers-NJMS (New Brunswick, NJ, USA), presented the preliminary data that indicated the profound effects of long COVID in ASD subjects who experienced a component of underlying neuroinflammation. Sars-Cov-2 activates signaling pathways through type 1 IFNs, creating neuropsychiatric features which are known to be seen in patients with primary immunodeficiency and cause dysregulated type 1 production or downstream signaling, often called interferonopathies. Non-ASD patients with long COVID exhibited predominant neuropsychiatric and neurological symptoms, and this appeared to be the case in ASD patients. However, long COVID may be under-diagnosed or under-treated in ASD subjects due to pre-existing difficult behaviors. Additionally, clinical features may be more complex, secondary to pre-existing neuroinflammation in some ASD subjects [14].

## 5. Industry Presentations

Seven industry presentations were opened by Sarika Agrawal, the Co-Founder of The BRAIN Foundation, where she presented her data about changing the status quo to help those who are diagnosed with ASD. Since ASD is deeply underfunded compared to many other neurological disorders, everyone needs to work together to directly influence the National Institute of Health’s plans for ASD research, as they have the largest funding for scientific research in the world.

Lynn Durham, CEO, and Baltazar Gomez-Mancilla, CMO of Stalicla (Geneva, Switzerland), talked about translating precision medicine for neurodevelopmental disorders. Stalicla created a Database Endophenotyping Patient Identification (DEPI) technology to match patients with neurodevelopmental disorders with tailored treatments by identifying the clinical and molecular characteristics of the patients. After receiving treatments, the patients had a significant negative correlation between the PDE4/3 inhibitor and gamma waves with a positive effect with an 8-point decrease in the SRS total score.

Katya Sverdlov, CEO, and Eugenia Steingold, CSO of Jelikalite (New York, NY, USA), talked about treating ASD with tPBM (transcranial photo biomodulation) based on new EEG evidence. tPBM treatment works because when it is applied, it increases the blood flow, increasing the oxygen level in the brain and producing more intracellular ATP. This causes cells to heal, reduces inflammation, and improves brain and functional connectivity in those with ASD. When they conducted a study, there was a significant reduction in symptoms in the childhood ASD scores, a significant decrease in delta waves, and an increase in the intensity of gamma waves. When used on patients, each patient had a unique positive response. For example, some started the treatment when they were non-verbal and began speaking short phrases after just two rounds of treatment.

Manish Arora (BDS, MPH, Ph.D.), CEO of the Linus Biotechnology Incorporation (New York, NY, USA), talked about a precision exposome biomarker platform for early detection and drug development in ASD. Using a single strand of hair, 2000+ data points determined the presence of certain molecules (non-genetic components), which could produce a molecular map of an individual over time. This platform currently has FDA Breakthrough designation for an at-birth diagnostic aid and is also being used in two Phase 2 drug trials. 

Robert Mills (Ph.D.), the Scientific Director at Precidiag Inc (Watertown, MA, USA), talked about how study factors have influenced the discrepancies found among researchers investigating ASD gut microbiomes. He showed that building machine learning models for diagnosing ASD from stools worked particularly well when models were built from data in China, which could suggest that there are geographical differences that play a role in autism. Their meta-analysis on reprocessed 16S ribosomal RNA gene amplicon (16S) sequencing data suggests that the gut microbiome can be altered in ASD patients [15].

Paul Song (MD), Vice Chairman and Senior Advisor at NKGen Biotech (Santa Ana, CA), talked about the regulatory role of natural killer (NK) cells in neuroinflammation and its potential therapeutic role in ASD. NKGen Biotech is aimed at growing natural killer (NK) cells and modifying NK cell receptors to increase binding and receptor expression, which can allow those who suffer from autoimmune diseases to increase the number of natural killer cells by receiving NK doses and increasing NK levels. One young adult, who had a rare case of Alzheimer’s, received five doses of NK cells and was able to significantly perform daily activities better.

Richard E Frye (MD, Ph.D.) discussed the importance of levoleucovorin created by Aprofol AG (Steinegg, Switzerland) and the ongoing randomized controlled clinical trials investigating this unique folate compound to treat ASD and identify biomarkers that can predict responses to this treatment. Many children with ASD test positive for antibodies to the folate receptor alpha, which impedes the transport of folate across the blood–brain barrier. He described his initial double-blind placebo control study, which demonstrated that leucovorin treatment for children with ASD and verbal communication impairment resulted in significant improvements in verbal communication.

Sean O’Sullivan, a prominent philanthropist from the O’Sullivan Foundation, talked during the conference dinner about kickstarting promising research areas and how high-net-worth individuals and family foundations can contribute to moving the field forward. He also emphasized the critical need for the work of the BRAIN Foundation to improve the outcomes and quality of life for individuals with autism.

## 6. Conclusions

The BRAIN Foundation’s annual conference, Synchrony, was held on the 2–4 of December 2022 this year. The conference hosted distinguished scientists and physicians who came together to present their findings and research on ASD in the hopes of working on and continuing to aid the ASD community by bringing more therapeutics to help those with NDDs. Synchrony 2022 covered a broad range of areas, from working on finding the important biomarkers of ASD to helping with early detection tools and working on basic science to evaluate various drug pathways and effects for treating the symptoms that affect their quality of life and outcomes. The successful Synchrony 2022 brought together the community of research experts, working groups, industry leaders, and the community to obtain more approved therapeutics and a standard of care to enhance the lives of ASD and NDD individuals.
